# A Parallel Coupled Lattice Boltzmann-Volume of Fluid Framework for Modeling Porous Media Evolution

**DOI:** 10.3390/ma14102510

**Published:** 2021-05-12

**Authors:** Hussein Alihussein, Martin Geier, Manfred Krafczyk

**Affiliations:** Institute for Computational Modeling in Civil Engineering (iRMB), TU Braunschweig, Pockelsstr. 3, 38106 Braunschweig, Germany; geier@irmb.tu-bs.de (M.G.); kraft@irmb.tu-bs.de (M.K.)

**Keywords:** lattice Boltzmann method, volume of fluid, dissolution, hydrated cement paste microstructures

## Abstract

In this paper, we present a framework for the modeling and simulation of a subset of physical/chemical processes occurring on different spatial and temporal scales in porous materials. In order to improve our understanding of such processes on multiple spatio-temporal scales, small-scale simulations of transport and reaction are of vital importance. Due to the geometric complexity of the pore space and the need to consider a representative elementary volume, such simulations require substantial numerical resolutions, leading to potentially huge computation times. An efficient parallelization of such numerical methods is thus vital to obtain results in acceptable wall-clock time. The goal of this paper was to improve available approaches based on lattice Boltzmann methods (LBMs) to reliably and accurately predict the combined effects of mass transport and reaction in porous media. To this end, we relied on the factorized central moment LBM as a second-order accurate approach for modeling transport. In order to include morphological changes due to the dissolution of the solid phase, the volume of fluid method with the piece-wise linear interface construction algorithm was employed. These developments are being integrated into the LBM research code VirtualFluids. After the validation of the analytic test cases, we present an application of diffusion-controlled dissolution for a pore space obtained from computer tomography (CT) scans.

## 1. Introduction

Reactive transport is related to a fundamental group of processes that shape the behavior of both natural and human-made artificial porous media. Within these processes, chemical, physical, and biological interactions occur over a wide range of temporal and spatial scales [1,2]. Examples of characteristic reactive transport problems are the storage of carbon dioxide in carbonate reservoirs [3,4], contaminated groundwater remediation [5], storage of radioactive waste [6], enhanced oil recovery [7], fuel cells [8], and the degradation of building materials [9].

The acceleration of the computations of large transient three-dimensional porous media microstructures is crucial, in order to be able to obtain the results in a reasonable amount of time. In this paper, we provided a detailed framework to model mass transport in porous media microstructures taking into account the morphological change due to dissolution. We explored the suitability of the factorized central moment (FCM) method, which demonstrated superior accuracy and stability over standard lattice Boltzmann models (LBMs) to simulate solute transport and morphological changes. The proposed framework was implemented to utilize a multi-core architecture, which included decomposing the computational domain into subdomains and using the message passing interface (MPI) for their communication. This feature allowed flexibility in choosing larger sizes of microstructure geometry to better represent the macroscopic properties of the porous media in the sense of a representative elementary volume (REV).

As an application, the framework was used to study diffusion-controlled dissolution problems from computed tomography images of hydrated cement pastes (HCPs). Previous initiatives to model physico-chemical phenomena at the pore scale [9,10,11,12,13] used microstructure modeling platforms (the so-called integrated kinetic models [14]), which simulate the hydration process itself to obtain virtual hydrated cement paste microstructures. Microstructure modeling platforms like CEMHYD3D [15] and HYMOSTRUC3D [16] were used to obtain the morphology of the cement paste at different hydration times. Although the advantage of such models may lie in their ability to provide accurate estimates of the hydrated phase fractions at specific degrees of hydration, their morphological features are not always correctly represented [17].

Moreover, previous similar frameworks or models used to model transport or dissolution [9,10,12,13] did not pay much attention to the precise position of the interface between the fluid and solid phases. Dissolution is a process characterized by propagating sharp fronts through the HCPs [18]. In order to precisely account for the resulting change in morphology, the volume of fluid (VOF) approach was used, through which it was possible to track the interface between the solid and void phases using the piecewise linear interface construction (PLIC) algorithm [19].

The reminder of the paper is organized as follows. In Section 2, the governing equations of the physicochemical system under consideration are summarized followed by a description of the factorized central moment lattice Boltzmann (FCM) method. The last part of the section briefly presents the volume of fluid (VOF) method and the phase interface construction using the piecewise linear interface construction (PLIC) method. In Section 3, we cover implementation details of the solute transport and morphology change algorithms as part of the simulation framework. In Section 4, we demonstrate the order of convergence of the FCM obtained by computing the error norm for a problem with Stefan moving boundary condition followed by a discussion of the preprocessing step required before using the 3D CT images in the simulations. Afterwards, the effective diffusivity in a 3D pore space reconstructed from CT images and, finally, calcium leaching are studied. The last part of the section is devoted to studying the parallel efficiency of the proposed scheme. Conclusions are provided in Section 5.

## 2. The Description of the Physicochemical Model

### 2.1. The Governing Equations

The formulation of reactive transport is divided into two main components: transport and reaction [20]. In weakly conductive porous media, transport processes are usually dominated by diffusion, while advection can occur if high flow velocities in the pores exist. Therefore, the so-called residence times for the chemical species inside the pores will vary spatially accordingly [2]. The other component, the reaction component, can be further divided into two types based on the phases or components to which the reactants belong; homogeneous and heterogeneous. In the case of homogeneous reactions, the reactants belong to the same phase or component. In the case of heterogeneous reactions, the reactants belong to different phases, for example gas and solid. Usually, heterogeneous reactions in porous media exist at the interface separating the solid matrix and the solution containing the chemical species inside the pores [21]. For heterogeneous reactions, the domain, over which the governing equations are supposed to be solved, changes as time passes. Hence, determining the domain of the fluid becomes an intrinsic part of the problem since it is unknown in advance [22].

Dissolution in porous media can be described as a diffusion-reaction problem. The diffusion equation or Fick’s second law is defined over a three-dimensional fluid domain $\Omega_{f}\subset R^{3}$:

(1) $$\partial_{t}\;c_{i}+\nabla \cdot \left(-D\nabla c_{i}\right)=R$$

where $c_{i}$ [$NL^{-3}$] is the concentration of the solute species *i*, *D* is the diffusion coefficient of the species *i* and has a dimension of [$L^{2}T^{-1}$], and *R* is a mass source term that describes the behavior of chemical reactions occurring inside $\Omega_{f}$. On the boundary ∂Ω, an appropriate boundary condition is prescribed to solve the above partial differential equation of a general form:

(2) $$Ac_{i}+B\nabla c_{i}\cdot n=C$$

where A, B, and *C* are three constants and n is the boundary unit normal vector. Applying different values to A, B, and *C* leads to different kinds of boundary conditions, mainly Dirichlet, Neumann, and Robin. They can be obtained by substituting the following values for the constants:
Dirichlet:A=1, B=0,$\;C=c{|}_{x=\partial \Omega }$Neumann:A=0, B=1,$\;C=-j/D{|}_{x=\partial \Omega }$Robin:$A=k_{c}$, B=−D,$\;C=kc_{l}^{eq}$
where *c* is a prescribed concentration, $c_{l}^{eq}$ is the saturation concentration in the fluid phase, j is a prescribed mass flux, and $k_{c}$ is the reaction rate constant.

### 2.2. Factorized Central-Moment Lattice Boltzmann Method for Modeling Mass Transport

In this work, the factorized central moment lattice Boltzmann method [23] was used for solving the advection-diffusion equation. The lattice Boltzmann method is derived from kinetic theory and is mainly applied for solving the Navier–Stokes equations, but other transport problems can be solved as well [24]. The LBM is based on a discrete velocity approximation for a velocity distribution function $f_{ijk}$ (when solving the Navier–Stokes equations) or $h_{ijk}$ (when solving the advection-diffusion equation) where i,j,k∈W are the integer quantum numbers of the three discrete velocity components. Here, a tensor product velocity set with i,j,k∈{−1,0,1} was used, leading to 27 discrete velocities. This particular choice of discrete velocities allowed for a specific time step Δt in which the distributions travel a discrete distance iΔx between two neighboring lattice nodes. Due to this arrangement, no interpolation of fluxes was required, and conservation laws were rigorously fulfilled in the simulation domain. However, at boundaries, this has to be enforced explicitly. After streaming, the particle distribution function undergoes collision on nodes. The collision operator has to enforce the particular transport equation, which is usually done by imposing specific conservation laws and fluxes via suitable equilibrium distributions and by choosing a particular relaxation rate to match the desired transport coefficients. This is often done directly on the distributions leading to the lattice Bhatnagar, Gross, and Krook (BGK) method [25].

The lattice BGK method is popular due to its simplicity, but suffers from considerable stability problems, in particular for small transport coefficients, and from accuracy deficits for large transport coefficients. The stability of the lattice BGK method can be improved by applying different relaxation rates for different moments leading to the class of multiple relaxation time (MRT) methods. In this approach, the distributions have to be transformed into an equivalent set of moments for the collision in moment space in which each moment is assigned its own time scale. Unfortunately, the transformation to moments is not unique. It can be reasoned that moments can evolve only on different time scales if they are independent observable quantities. MRT models are usually designed around a static transformation matrix, which can impose the independence of different moments in equilibrium at best. The factorized central moment method [23] obtains an approximation of the independence of the moments through the factorization of the equilibrium moments according to their cardinal directions displaced by the mean flow (hence, central moments). By doing so, the transformation itself becomes highly non-linear.

Starting from the definition, the central moments are obtained by the following factorization [24]:

(3) $$\begin{array}{ccc} M_{000} & = & k_{000} \end{array}$$

(4) $$\begin{array}{ccc} M_{\left(100\right)} & = & k_{\left(100\right)} \end{array}$$

(5) $$\begin{array}{ccc} M_{\left(110\right)} & = & k_{\left(110\right)} \end{array}$$

(6) $$\begin{array}{ccc} M_{111} & = & k_{111} \end{array}$$

(7) $$\begin{array}{ccc} M_{\left(200\right)} & = & k_{\left(200\right)}-\frac{1}{3}k_{000} \end{array}$$

(8) $$\begin{array}{ccc} M_{\left(210\right)} & = & k_{\left(210\right)}-\frac{1}{3}k_{\left(010\right)} \end{array}$$

(9) $$\begin{array}{ccc} M_{\left(220\right)} & = & k_{\left(220\right)}-\frac{1}{3}k_{000} \end{array}$$

(10) $$\begin{array}{ccc} M_{\left(221\right)} & = & k_{\left(221\right)}-\frac{1}{9}k_{\left(001\right)} \end{array}$$

(11) $$\begin{array}{ccc} M_{\left(211\right)} & = & k_{\left(211\right)}-\frac{1}{3}k_{\left(011\right)} \end{array}$$

(12) $$\begin{array}{ccc} M_{222} & = & k_{222}-\frac{1}{27}k_{000} \end{array}$$

where the brackets indicate permuting the index to obtain the other moments, i.e., $k_{\left(200\right)}$ stands for $\left(k_{200},k_{020},k_{002}\right)$. The factorized central moment method was introduced to solve the Navier–Stokes equations [23]. However, it can also be used to solve the advection-diffusion equation where the only conserved moment is the zero-order moment, which is equal to the mass density $M_{000}=\rho$. The diffusion coefficient is then [24]:

(13) $$D=\frac{1}{3}\left(\frac{1}{\omega_{100}}-\frac{1}{2}\right)\frac{\Delta x^{2}}{\Delta t}$$

For the D3Q27 lattice, the transformation of the solute distributions $h_{ijk}$ into central moments is presented in Appendix A. The collision is realized by the following equation, where all equilibria of the factorized central moments $M_{nrs}$ are zero [24]:

(14) $$M_{nrs}^{*}=\left(1-\omega_{nrs}\right)M_{nrs}$$

where the sum of the indices n, r, and *s* indicates the order of the moment and $\omega_{nrs}$ is the relaxation rate corresponding to the moment $M_{nrs}$. From Equation (13), we can see that the diffusivity is determined using only one relaxation rate $\omega_{100}$. The rest of the relaxation rates were set to unity [24]. In order to obtain the post collision solute distributions $h_{ijk}^{*}$, we used the backward transformation described in Appendix A. This collision step is followed by the usual streaming step.

A particular advantage of the FCM over the classical MRT method is the Galilean invariance of the moment transformation, which naturally includes advective transport. In the current study, however, this property was not used as we investigated diffusion-dominated transport.

Appropriate kinetic boundary conditions are required to model their macroscopic counterparts defined in Equation (2). Here, single-node boundary conditions were used according to [24,26]. Dirichlet boundaries were used in case of diffusion-controlled dissolution [27]. The missing distributions are obtained from:

(15) $$h_{\bar{i}\bar{j}\bar{k}\left(t+\Delta t\right)}=h_{ijkt}^{eq}\left(c_{l}^{eq}\right)\;+\;h_{ijkt}-h_{ijkt}^{eq}\left(c_{l}\right)$$

where $h_{ijkt}^{eq}$ is the BGK equilibrium distribution, $h_{ijkt}$ is the post collision distribution in the direction of the solid phase, $c_{l}^{eq}$ is the saturation concentration in the fluid phase, and $c_{l}$ is the actual concentration at the boundary node.

For the Neumann boundary, the unknown distribution $h_{\bar{i}\bar{j}\bar{k}\left(t+\Delta t\right)}$ entering the domain from the boundary at the next time step can be obtained from linear interpolation between the boundary distribution and the post collision distribution moving in the opposite direction. More details about implementing this boundary condition were provided in [26]. The missing distribution can be identified as:

(16) $$h_{\bar{i}\bar{j}\bar{k}\left(t+\Delta t\right)}=\frac{\left(q-1\right)\left(h_{ijkt}^{*}-\omega h_{ijkt}^{eq}\right)}{\left(q+1)(\omega -1\right)}+\frac{q\left(h_{ijkt}^{*}+h_{\bar{i}\bar{j}\bar{k}t}^{*}\right)-6w_{ijk}\left(e_{ijk}\cdot j\right)}{q+1}$$

where *q* is the subgrid distance from a boundary grid node to the actual position of the interface, j is the imposed flux on the boundary, $e_{ijk}$ is the link direction, and $w_{ijk}$ is the weight for the corresponding link. Setting the flux j to zero implies a no-flux boundary condition.

### 2.3. Volume of Fluid with Piecewise Linear Interface Construction

The main challenge in simulating dissolution as in any other multiphase problem is to handle the changes occurring on each side of the interface between different phases. For a dissolution problem, we assumed that no transport occurs within the solid phase. Mass transport only occurs across the interface, resulting in changing the morphology of the solid phase boundary location. In order to capture this change, the VOF-PLIC approach was employed, where the volume fraction of one phase is traced over the simulation time.

The standard VOF method depends on solving an additional transport equation of the so-called fill level ϵ, defined as the ratio of the volume fraction of a phase *V* to the volume of a specific control volume $V_{cv}$ [28]. The control volume, also called the cell, takes either a uniform or nonuniform polygonal shape in 2D or polyhedral in 3D. Usually, the method is composed of two steps. In the first step, the interface reconstruction step, the location of an interface is obtained from the fill volume information. The second step is the application of an advection rule of the fill volume information.

The fill volume values are mapped to the interval [0, 1]. In our case, the phase under consideration was the reactive solid phase. Therefore, a cell of fill volume ϵ=1 is considered a solid cell, while a cell of fill volume ϵ=0 is considered a fluid cell, and a cell of a fill level in between is considered as an interface cell. In the LBM, the lattice spacing is usually taken as Δx=1, making $V_{cv}=1$ and, therefore, ϵ = V = 1.

In case of a multiphase problem of phases that are mainly composed of a solid and a fluid phases, the interface is transported by the kinetics of dissolution. Thereby, the second step of the VOF, i.e., the advection step, does not depend on solving the Navier–Stokes equations as in the standard VOF.

Several methods for obtaining the position of the interface within a cell using the fill volume information were introduced earlier. Among the first is the simple line interface calculation (SLIC), which uses a reconstruction scheme that results in interfaces aligned with the cell face [29]. A more accurate representation of the interface is Youngs’ piecewise linear interface construction (PLIC) [30], where the normal vector of the interface is calculated using the fill volume field and employed in the reconstruction of the interface. This method results in representing the phase interface as a set of discontinuous plane segments. Puckett [31] followed by Pilliod [32] introduced second-order schemes for evaluating a surface interface normal from the discrete fill level field. Furthermore, higher order interface representation methods were proposed, e.g., by Ginzburg and Wittium [33] where a cubic spline was used.

**Figure 1:**
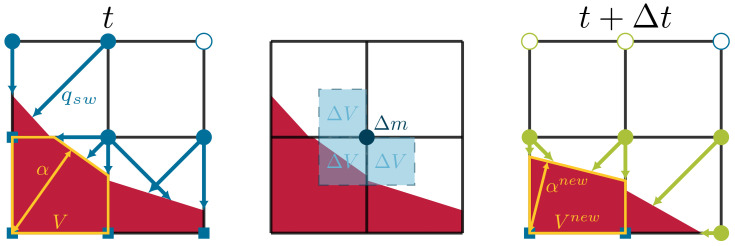
Schematic of the solid fluid interface and the reconstruction of the interface contour. ● is a boundary fluid node; ■ is a solid node; and ○ is the fluid node.

In the PLIC method, the interface is represented as a line segment in 2D or a plane in 3D. In 3D, for a plane to be uniquely defined, both its normal n and the shortest distance α from the origin of a frame of reference to the plane are required, as seen in Figure 1. Hence, the plane is defined by the equation:

(17) x·n = α

where x are the coordinates of all points in space forming the plane. In the LBM, we used a uniform, rectangular discretization of the space resulting in a grid of nodes. We defined the cells as the space between eight nodes of the LBM grids with one node on each corner of the cell. Therefore, the cells have the form of a cube. In addition, we used the edge length of Δx = 1. When a plane cuts the unit cell, a volume *V* is reserved underneath the plane. The value of *V* depends on $n\;=\{n_{1},n_{2},n_{3}\}$ and α. Its geometry takes the form of a polyhedral shape. To calculate *V* from the following equation, the normal components are rendered positive and sorted such that $n_{1}\leq n_{2}\leq n_{3}$:

(18) $$V\left(\alpha ,n\right)=\frac{\left(\alpha^{3}+\sum_{j=1}^{3}H\left(\alpha -\alpha_{\max}+n_{j}\right)\left(\alpha -\alpha_{\max}+n_{j}\right){}^{3}-\sum_{j=1}^{3}H\left(\alpha -n_{j}\right)\left(\alpha -n_{j}\right){}^{3}\right)}{6n_{1}n_{2}n_{3}}$$

where H is the Heaviside function and $\alpha_{\max}=n_{1}+n_{2}+n_{3}$. In Appendix B, the expanded form of Equation (18) is shown.

Since our aim was to determine the exact position of the plane, from Equation (17), we needed to know in advance both the normal information n and the parameter α. In PLIC, the normal estimation is usually achieved by evaluating the gradient of the fill level field.

The normal to the interface can be determined using an approximation of the gradient by applying central divided differences on the fill level values at each interface cell in the fill level field.

(19) $$n=-\frac{\nabla V}{\left||\nabla V|\right|}$$

The approximation reads:

(20) $$\nabla V=\frac{1}{2\Delta x}\left(\begin{array}{c} {\bar{\epsilon }}_{x}\left(x+\Delta x\;\hat{i}\right)-{\bar{\epsilon }}_{x}\left(x-\Delta x\;\hat{i}\right)\\ {\bar{\epsilon }}_{y}\left(x+\Delta x\;\hat{j}\right)-{\bar{\epsilon }}_{y}\left(x-\Delta x\;\hat{j}\right)\\ {\bar{\epsilon }}_{z}\left(x+\Delta x\;\hat{k}\right)-{\bar{\epsilon }}_{z}\left(x-\Delta x\;\hat{k}\right) \end{array}\right)$$

where ${\bar{\epsilon }}_{i}\left(x\right)$ is the average value of the neighboring cells’ fill level at x. The interface cell is located at the center of a block of 3×3×3 cells and surrounded by 26 neighboring cells. Hence, the average ${\bar{\epsilon }}_{i}\left(x\right)$ can be obtained from:

(21) $${\bar{\epsilon }}_{x}\left(x,y,z\right)=\sum_{i=-1}^{1}\sum_{j=-1}^{1}V\left(x,y+i,z+j\right).w\left(i,j\right)$$

(22) $${\bar{\epsilon }}_{y}\left(x,y,z\right)=\sum_{i=-1}^{1}\sum_{j=-1}^{1}V\left(x+i,y,z+j\right).w\left(i,j\right)$$

(23) $${\bar{\epsilon }}_{z}\left(x,y,z\right)=\sum_{i=-1}^{1}\sum_{j=-1}^{1}V\left(x+i,y+j,z\right).w\left(i,j\right)$$

If the normal estimation is required for a cell that is located on a boundary where Equation (20) cannot be applied due to having a neighbor position located outside the domain, the central difference is replaced by the forward or backward difference. A boundary cell may lie either on a plane, an edge, or a corner. For each of the previous cases, a smaller block was used to calculate the average fill volume. In the case of a cell located on a plane, a block of 18 cells was used, while for a cell located at an edge, a block of 12 cells was used, and for a cell located at a corner, a block of eight cells was used. For these special cases, we used the center of mass method [34,35] for the weights *w* in Equations (21)–(23).

The accuracy of the estimation of the normal depends on the weighting factor *w*. Using different values for w(i,j) results in different isotropy properties. The approach known by the center of mass [34,35] uses a value of w=1/18. Parker and Youngs [36] suggested the use of the following weighting factors:

(24) w(0,0)=1/8,w(±1,0)=w(0,±1)=1/16,w(±1,±1)=1/32

The method of Parker and Youngs [36] was found to be more accurate than the center of mass method in [37]. However, neither method was observed to reach second-order convergence. In the actual implementation, the weights were scaled up so that they became integers as we were only interested in the direction of the normal, and any constant was eliminated by the normalization in Equation (19). Using integer weights avoids unnecessary round-off errors. The convergence order of the finite difference is independent of the weights. However, the isotropy is not. While we used the weights of Parker and Youngs in this study, we note here that these weights did not lead to an isotropic leading order error. Using lattice Boltzmann weights for the D3Q27 lattice would have this property, as we explain in Appendix C.

If higher accuracy is required, optimization methods like the least-squares VOF interface reconstruction algorithm (LVIRA) [31] or its efficient counter part (ELVIRA) [32] can be employed. Each time a normal needs to be estimated, the former method solves an optimization problem, which is computationally expensive, while the latter method requires a larger cell stencil, which adds an additional complexity to the parallel implementation.

After estimating the normal in an interface cell, the plane parameter α is the only parameter to be determined. Usually, the interface reconstruction step is conducted after evaluating the fill level field in all cells, and hence, the fill volume *V* information is available. By taking the inverse of Equation (18) using the normal information, one can determine the plane parameter α(V,n).

The inverse is achieved first by sorting the estimated normal n components as follows $0<n_{1}\leq n_{2}\leq n_{3}\leq n_{1}+n_{2}\leq n_{1}+n_{3}\leq n_{2}+n_{3}$ or $0<n_{1}\leq n_{2}\leq n_{1}+n_{2}\leq n_{3}\leq n_{1}+n_{3}\leq n_{2}+n_{3}$, which results in having Equation (18) as a piecewise function of the independent variable α defined over seven distinct intervals. Afterwards, the inverse of each sub-function is calculated. The procedure for calculating the inverse is shown in Appendix B.

## 3. Implementation Details

**Figure 2:**
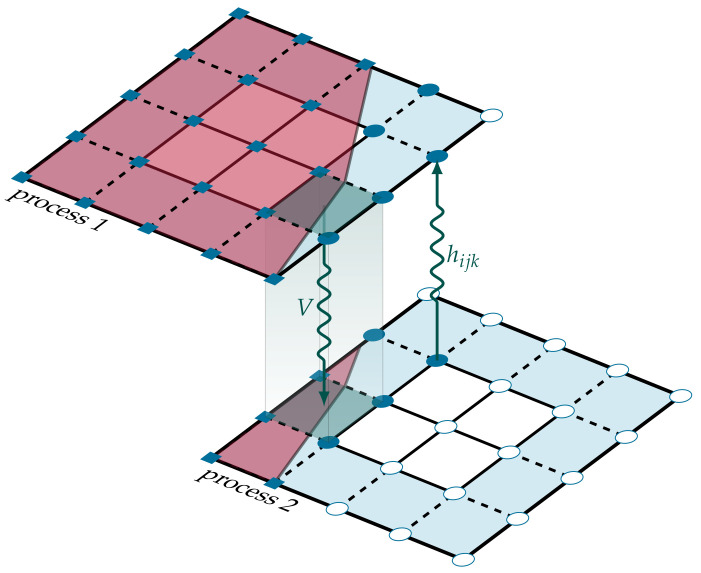
Two neighboring VirtualFluids blocks belonging to different processes/subdomains. Within each time step, both blocks exchange information.

Both the FCM and the VOF-PLIC were implemented in VirtualFluids [38], which is a parallel framework for conducting fluid simulations. To reduce the computational time, the coupled algorithm was run in parallel, where the computational grid containing the LBM nodes and the VOF cells was decomposed into smaller objects, called blocks. These blocks were distributed among different computing processes (see Figure 2). A block in VirtualFluids acts as a superset containing different data structures for the particle distributions, cell state, fill volume information, and normal vector information. Within one iteration of the framework algorithm, several synchronization points exist, where information is exchanged between the blocks, as seen in Figure 2. Since not all parts of the domain might be used during the computations, blocks can be activated where needed and deactivated where not needed, for example in non-reactive solid zones. To study the parallel performance of the coupled LBM-VOF-PLIC scheme, scalability tests results are shown and discussed in Section 4.3.

The coupling between VOF-PLIC and LBM occurs after applying the boundary conditions. From Equation (15), in the case of diffusion-controlled dissolution the distributions $h_{\bar{i}\bar{j}\bar{k}\left(t+\Delta t\right)}$ that entered the domain are calculated. As a result, the macroscopic change in mass at the grid node where the boundary condition was applied is calculated from:

(25) $$\Delta m=\sum_{ijk}h_{\bar{i}\bar{j}\bar{k}\left(t+\Delta t\right)}-h_{ijk}$$

The change in mass is transformed into a change in volume using Equation (26). This change in volume ΔV is to be subtracted from the existing solid content of the cells surrounding the boundary node, as shown in Figure 1.

(26) $$\Delta V=\frac{1}{N}\frac{\Delta m}{c_{s}^{eq}-c_{l}^{eq}}$$

where *N* is the number of interface cells, i.e., partially filled cells neighboring a boundary node.

Updating the cells involves either updating the reactive solid volume in a cell or updating both the state of the cell and its reactive solid volume. The updated volume of each interface cell $V^{new}$ is obtained by subtracting the volume of solid calculated from Equation (26) from the actual volume of solid *V* that the cell contains. The volume after the update becomes $V^{new}=V-\Delta V$. If $V^{new}>0$, only the new volume is mapped to the cell, and its interface state persists. On the other hand, if $V^{new}<0$, the cell state is set to fluid, and its volume is set to zero. Afterwards, a search for a donor solid cell is conducted with the help of the interface normal. Since the interface normal is pointing towards the fluid domain, its inverse direction is used to find all solid cells in the domain composed of the direct next neighbors. If a solid cell is found, the new volume after the update becomes $V^{new}=1-\Delta V$, and the new state of the cell becomes interface. The updated volume and the position of the new interface cell are registered in a container called the fresh interface cells set. This step is conducted in order not to invalidate the state of the solid cell, which may have been sought by another neighboring cell.

Changing the state of the cells is reflected on the state of the nodes. Node updating is more complex than the updating of cells, as the node holds more information than the cell. We can split the information residing on a node into two groups: the first group is related to geometric information, and the second is related to the distributions. The geometric information update includes calculating the new sub-grid distances *q* due to the change in the position of the interface. The distributions is only affected in the case of uncovering solid nodes, which results in having new fluid nodes, i.e., solid nodes converted into boundary fluid nodes, where new distributions must be initialized. These new fluid nodes are initialized using the equilibrium distributions of a concentration value equal to that of the fluid saturation concentration $c_{l}^{eq}$.

## 4. Results and Discussion

### 4.1. Channel with Moving Interface: Diffusion Controlled

We simulated here a Stefan moving boundary problem of a semi-infinite space. In this case, the dissolution was diffusion-controlled, meaning that the reaction was assumed to be instantaneous. The interface concentration was always at saturation $c_{i}=c^{eq}$. The analytic solution for the interface position is given in Equation (27) and for the concentration profile in Equation (28) [39].

(27) $$r-r_{0}=2\lambda \sqrt{Dt}$$

where $r_{0}$ is the initial position of the interface and r is the position of the interface after a time *t*.

(28) $$c\left(x,t\right)=c^{eq}\frac{\mathrm{erfc}\left(\frac{x-x_{0}}{2\sqrt{Dt}}\right)}{\mathrm{erfc}\left(-\lambda \right)}$$

with λ obtained after solving the following transcendental equation:

(29) $$\sqrt{\pi }\;\lambda \;e^{\lambda^{2}}\;\mathrm{erfc}\left(-\lambda \right)=\frac{c^{eq}-c_{0}}{c_{s}-c^{eq}}$$

**Figure 3:**
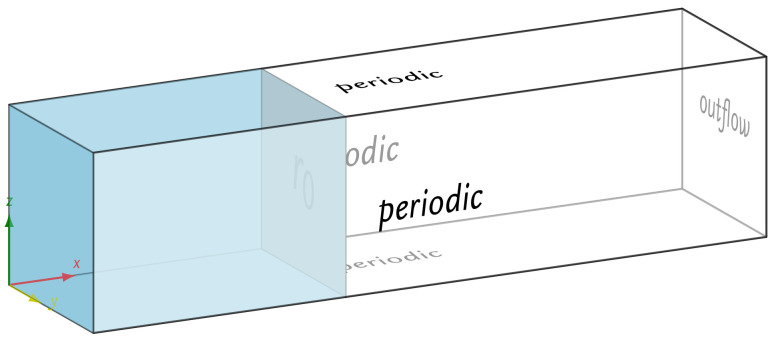
Geometry setup for the simulations in Section 4.1.

Several cases were studied using the following values of the ratio of saturation concentrations in fluid and solid phases $c^{eq}/c_{s}=\left\{0.1,0.3,0.4\right\}$. The initial concentration was set to $c_{0}=c\left(x,0\right)=0$. The diffusion coefficient was assumed to be $D=10^{-9}$$\frac{m^{2}}{s}$. Different resolutions were used in the simulations Δx={0.025,0.05,0.1} mm. The geometry used in this test was a channel of length l=10 cm extending along the *x* axis and width and height of w=h=2 cm. The geometry was split into two phases: the solid phase extended in the interval [0,2.5] cm, and the rest of the channel was occupied by the fluid phase. Periodic boundary conditions were applied on the faces perpendicular to the *y* and *z* directions, as seen in Figure 3. A constant concentration $c^{eq}$ boundary condition was applied at the interface of the channel. The simulation time interval was [0, 20,000] s. The lattice Boltzmann diffusivity used in the simulations was $\tilde{D}=0.01\;\frac{\Delta x^{2}}{\Delta t}$.

**Figure 4:**
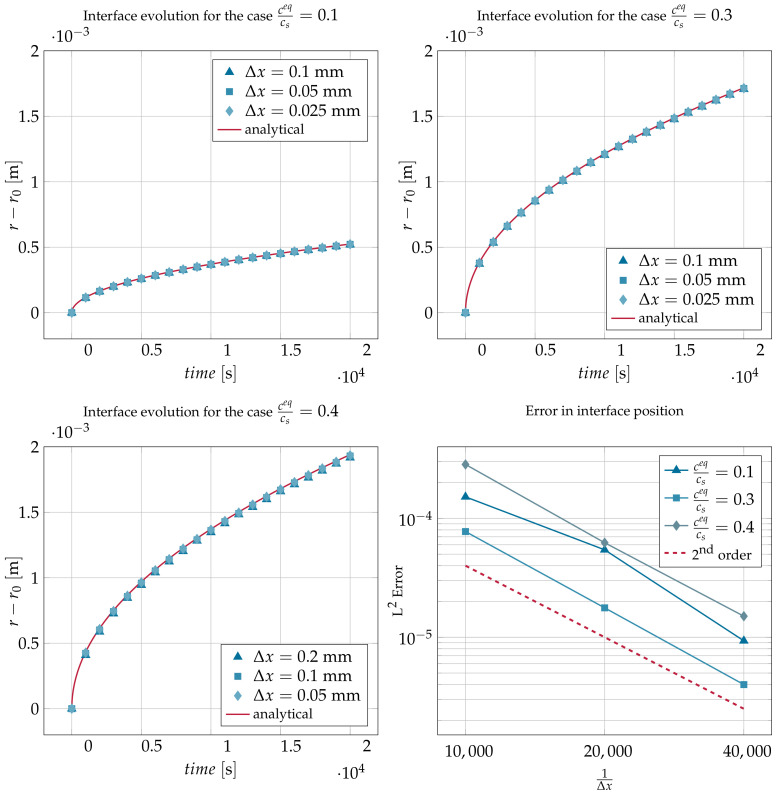
Comparison of the analytic solution and the simulated results for the position of the interface in the case of diffusion-controlled dissolution for different solid/fluid saturation ratios and for different grid resolutions together with a plot showing the convergence of the $L^{2}$ norm of the error in the interface position.

After running the simulations using the previous parameters, the mean position of the points composing the interface is plotted against time in Figure 4. A very good agreement was obtained between the analytic and the simulation results. The grid convergence study in the same figure shows second-order convergence. The error in interface-position is calculated from the following $L^{2}$ norm:

(30) $$L^{2}=\frac{1}{N}\sqrt{\sum_{i=1}^{N}{\left(\frac{r_{i}^{simulation}-r^{analytic}}{r^{analytic}}\right)}^{2}}$$

where *N* is the total number of mesh points in the interface, $r_{i}^{simulation}$ is the position of node *i* in the interface obtained from the simulation, and $r^{analytic}$ is the position of the interface calculated from Equation (27).

In real applications, the resolution required to capture small features like capillary pores sometimes reaches a magnitude of micrometers. This implies a dramatic reduction of the length of the time step. To feasibly simulate these systems, the simulations needs to be accelerated. This is achieved through increasing the diffusivity $\tilde{D}$ in the pore water. Increasing diffusivity results in decreasing the number of time steps required for the simulation.

Here, three examples illustrate the effect of using rescaled (increased) diffusivity $\tilde{D}$ values on accuracy. The same domain of the previous example was used. The resolution used in these simulations was Δx=1μm. The chosen ratio of saturation concentrations in the fluid and solid phases was $\frac{c^{eq}}{c_{s}}=0.001$. The simulations were run for about 55,000 Δt.

**Figure 5:**
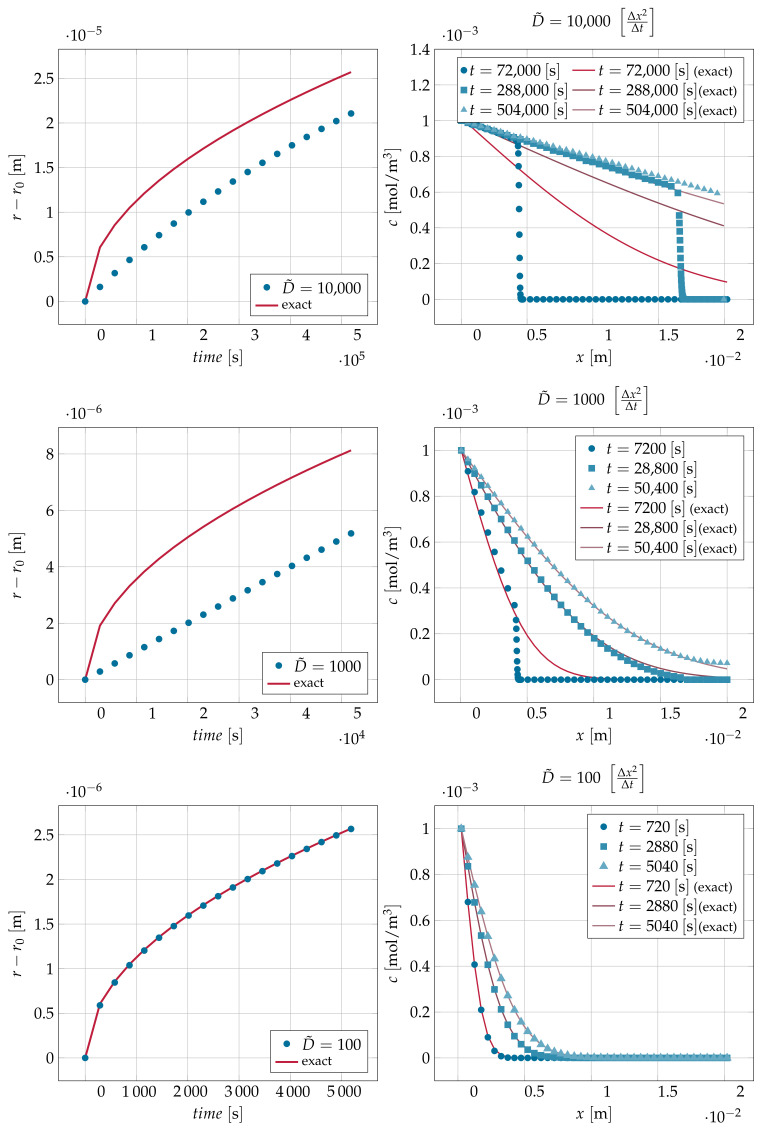
The interface displacement predicted numerically for the channel with diffusion-controlled dissolution using three different values for $\tilde{D}\in \left\{100,1000,10,000\right\}\;\frac{\Delta x^{2}}{\Delta t}$ together with the exact displacement calculated from Equation (27) (left column). The concentration profiles corresponding for different diffusivities $\tilde{D}$ compared with the exact profiles for different times are shown in the right column.

As shown in Figure 5, the error in the concentration for the cases of diffusivity of $1000\;\frac{\Delta x^{2}}{\Delta t}$ and $10,000\;\frac{\Delta x^{2}}{\Delta t}$ had severe consequences on the dynamics of the interface position, where the position of the interface lagged behind its analytic counterpart. On the other hand, decreasing the diffusivity to $\tilde{D}=100$ in the third case led to an excellent match with the analytic solution for both the concentration profile and the interface position.

For cases of diffusivity of $1000\;\frac{\Delta x^{2}}{\Delta t}$ and $10,000\;\frac{\Delta x^{2}}{\Delta t}$, it was observed that a steep front of concentration was formed in the domain. The speed of sound of the current LBM was $C_{s}=\frac{1}{\sqrt{3}}\;\frac{\Delta x}{\Delta t}\approx 0.577\;\frac{\Delta x}{\Delta t}$. The analytic solution was derived from Fick’s law in which the speed of sound and hence ballistic transport were absorbed into the diffusion coefficient *D*. The analytic solution can outrun the speed of sound because it does not depend on the speed of sound in the first place. The LBM, on the other hand, is bound by its kinetic heritage to obey the principle that diffusive transport cannot be faster than ballistic transport.

In what follows, the diffusion velocity formulation is used for analysis purposes. The diffusion velocity transformation was originally introduced to provide stability for advection dominated problems [40,41]. In the case of solving for diffusion without advection (v=0), the diffusive flux −D∇c can be transformed into an equivalent advective flux of $u_{d}c$, where $u_{d}$ is the diffusion velocity. A front was formed when the diffusion velocity $u_{d}=\frac{-D\nabla c}{c}$ exceeded the speed of sound of the method $C_{s}$. Table 1 shows the positions in the domain where the diffusion velocity $u_{d}$ reached the speed of sound for the first two cases in Figure 5. The front location was close to the position where the diffusion velocity exceeded the speed of sound. If the diffusion velocity was everywhere smaller than the speed of sound no front was formed, and Fickian behavior was observed.

### 4.2. Transport and Morphology Change of Tomographic Images of Cement-Based Materials

Ideally, the methodology to assess the morphology of the internal pore structure would have certain goals according to [42]. Any investigation within this ideal methodology is supposed to be: three-dimensional, non-destructive, free of constraints applied during preparation, free of hypothesis or assumptions on its pore geometry (directly measured), and having a high resolution to detect all pores inside the cement paste at all scales (micro and nano) [42]. Most of these requirements are provided by X-ray computed tomography (CT), making it a suitable candidate to study transport processes in cement paste at the pore scale.

#### 4.2.1. Preprocessing of the CT Scans

In order to use the VirtualFluids framework to study transport in realistic hydrated cement paste microstructure models, we used a data set provided by the National Institute of Standards and Technology (NIST), Visible Cement Dataset [43]. The cement used in preparing the specimens was the Cement and Concrete Reference Laboratory (CCRL) cement 133. The physical and chemical properties of cement 133 are listed in the NIST Cement Images database [44].

**Figure 6:**
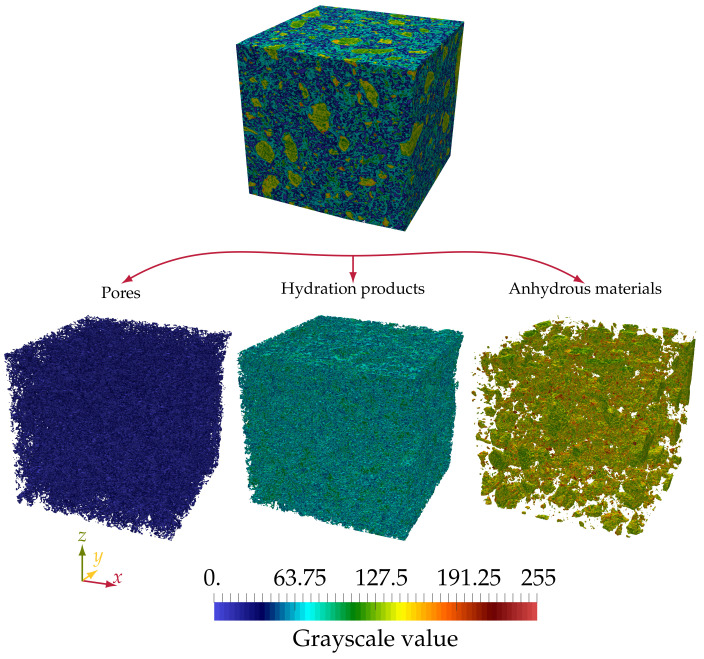
The hydrated cement sample pt045_sld_7dv1c300 [43,44]. A core cube of dimensions $300\times 300\times 300\;\mu m^{3}$ was taken as a representative element volume. Three phases (pores, hydration products, and unhydrated cement) were obtained using Power’s model [45] (Equation (31)).

The NIST data set contains 3D images of cement pastes of water/cement (w/c) ratios between 0.3 and 0.45 taken at different hydration times. The samples were analyzed by a 3D microtomography device at the European Synchrotron Radiation Facility (ESRF). The end-product of the imaging process was a 3D voxel set with a voxel size of 0.95μm. The whole sample contained $1024^{3}$ voxels. Bentz et al. extracted a smaller cubic sub-sample (see Figure 6) that contained $300^{3}$ voxels [43].

Each voxel in the 3D image was assigned a value between zero and 255, known as the grayscale value. These values are proportional to the linear attenuation coefficient of the material contained in the voxel. Materials that have higher densities, for example unhydrated cement, appear brighter while areas with lower densities appear darker [43].

In order to obtain the pore space of the cubic sub-sample, we conducted a histogram-based segmentation using Power’s model [45]:

(31) $$\begin{array}{cc} \theta_{pores} & =\frac{w/c-0.36\alpha }{w/c+0.32}\\ \theta_{unhydrated} & =\frac{0.32(1-\alpha )}{w/c+0.32}\\ \theta_{hydrated} & =1-\theta_{pores}-\theta_{unhydrated} \end{array}$$

**Figure 7:**
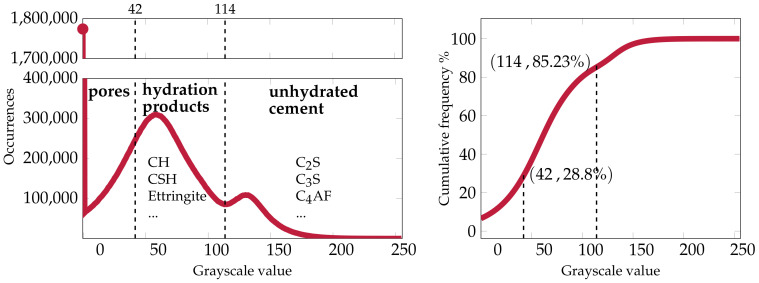
The histogram of the CT sample is segmented into three different phases: pores, hydration phases and unhydrated cement using Powers [45] model (Equation (31)) (**left**). The cumulative frequency of the distribution is shown on the right.

The histogram of the sample is shown in Figure 7.

Here, we followed the thresholding described in [46], where the threshold between the hydrated and unhydrated products was $T_{1}=114$. This allowed calculating the unhydrated cement volume fraction to be $\theta_{unhydrated}=0.154$. Then, from Equation (31), we can calculate the level of hydration α=0.63. From the previous, we can calculate the volume fractions of the hydration products and the pores to be $\theta_{hydrated}=0.558$ and $\theta_{pores}=0.288$, respectively. The final pore space, hydration products, and unhydrated cement after thresholding are depicted in Figure 6.

In this paper, all our simulations used a particular data set (i.e., pt045_sld_7dv1c300 [44]) which had a water/cement ratio of w/c=0.45 and a hydration time of 137 h. The reason for using this particular data set was that it was the one with the longest hydration time among the available samples.

#### 4.2.2. Diffusion through Capillary Pores in HCP Microstructures

Diffusion of a solute inside the pores of the HCP microstructures was studied to obtain the effective diffusivity, which is considered to be an important factor in determining the service life of the structure [47]. The effective diffusivity is a macroscale property of porous media that depends on several factors: the porosity of the medium, its morphology, and the diffusivity of the species itself in pure water [48].

Various experimental methods have been proposed to measure the effective diffusivity in HCP. In addition, several attempts to tackle the problem through modeling have been developed. The state-of-the-art of different experimental and numerical techniques used to determine the effective diffusivity can be found in Patel’s work [9].

The computational procedure to calculate the effective diffusivity included solving the diffusion equation without advection in the fluid domain of the microstructure, which is composed of the pore phase, as shown in Figure 6. The other two phases, i.e., the hydration products and the unhydrated cement, were considered as non-diffusive. Patel [9] collected experimental data for the diffusivity in the calcium silicate hydrate (CSH) phase and found it to be more than two orders of magnitude smaller than the diffusivity in the pore space. A discrepancy was observed between diffusion measurements based on electrical resistance and based on diffusion [49]. Electrical resistivity measurements might overpredict the effective permeability by including the porosity of the CSH phase, which has no significance for the actual diffusion such that neglecting the diffusion through the hydration products was justified in our simulations [46].

Three simulations were conducted to study the magnitude of the effective diffusivity of a solute diffused along a different main axis of the sample. In the first simulation, no transformation was applied on the microstructure shown in Figure 6, and the inlet and outlet were located on the planes x=0Δx and x=300Δx. In the second simulation, a clockwise rotation of $90^{\circ }$ about the *z* axis was applied, and the inlet and outlet were located on the planes y=0Δx and y=300Δx. In the last case, the microstructure was rotated about the *y* in the counter-clockwise direction by $90^{\circ }$ where the inlet and outlet were located on the planes z=0Δx and z=300Δx.

At the inlet, we assigned a constant concentration of one for the three cases, while the outlet was assigned a constant concentration of zero. Apart from the inlet and the outlet, the walls surrounding the sample in other directions each were assigned a no-flux boundary condition. Moreover, for both of the non-diffusive phases, we assigned a no-flux boundary condition, respectively. The diffusivity used in the simulations was $\tilde{D}=1\;\frac{\Delta x^{2}}{\Delta t}$, corresponding to a physical diffusivity of $D=10^{-9}\;\frac{m^{2}}{s}$, and the simulations were run for 1,000,000Δt. The simulations were run until the steady state had been reached, which was assumed to be the case when the difference in the total sum of the normal to the wall component of the mass flux at the outlet was less than $10^{-3}$ over 100,000Δt.

**Figure 8:**
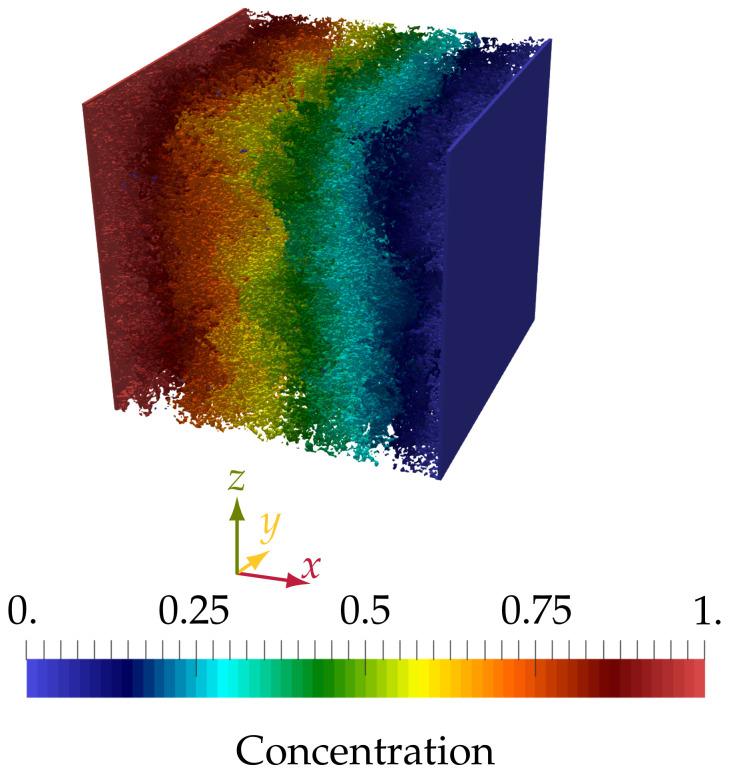
The concentration field of the solute after reaching the steady state.

According to [50], the effective diffusivity $D_{e}$ can be calculated from:

(32) $$D_{e}=\frac{\int_{outlet}\;j_{D}\cdot n}{A\Delta c}$$

where n is the normal to the outlet (in the opposite direction of the domain), Δc is the difference between the inlet and outlet concentration, and *A* is the area of the outlet. After reaching the steady state, Equation (32) is used to calculate $D_{e}$. The total sum of the normal to the wall component of the mass flux at the outlet and the calculated $D_{e}$ for each simulation is shown in Table 2. Figure 8 shows the concentration field of the solute after reaching the steady state.

Garboczi and Bentz [51] proposed a model described in Equation (33) that is calibrated against experiments to obtain $D_{e}$.

(33) $$\frac{D_{e}}{D_{0}}=0.001+0.07{\theta_{pores}}^{2}+1.8H\;\left[\theta_{pores}-0.18\right]\;{\left(\theta_{pores}-0.18\right)}^{2}$$

where H is the Heaviside function and $\theta_{pores}=0.288$, as obtained earlier (see Figure 7). By direct comparison of our results to the above formula, as shown in Table 2, we found a good agreement in the third case, where the gradient of the solute was along the *z* axis. In the other two cases, we observed that our model overestimated the value of $D_{e}$, although the CSH phase was considered non-diffusive.

The discrepancy between the simulation results and the values obtained from Equation (33) could be related to the explanation offered by Karim and Krabbenhoft [46]. They suggested that the overestimated $D_{e}$ values are due to the threshold level between pores and solid materials of 42 being too high. As a result of this thresholding, a voxel belonging to the pores may actually contain a solid that would have contributed to the resistance against diffusion. On the other hand, decreasing the threshold to capture the correct effective diffusivity results in having lower porosity that does not correspond to the w/c of the microstructure.

#### 4.2.3. Simulating Calcium Leaching in HCP Microstructures

Dissolution in cementitious materials is the process of releasing ions from the solid hydrated products, mainly calcium. The solid phases that are mainly responsible for releasing calcium are the portlandite (CH) and calcium silicate hydrate (CSH) [52,53,54,55]. The calcium ions are then transported mainly by diffusion in the saturated pores [56,57,58]. The combination of the two processes is referred to as calcium leaching. According to their significance in the hydrated cement paste, the hydrated phases containing calcium are calcium silicate hydrate (CSH), portlandite (calcium hydroxide) (CH), and other phases collectively known as AFm, resulting from the hydration of aluminates and ferrites. The following chemical equations are the assumed reactions taking place to produce the hydrates [59,60,61]:

(34) $$2\;C_{3}S+10.6\;H\to C_{3.4}S_{2}H_{8}+2.6\;CH$$

(35) $$2\;C_{2}S+8.6H\to C_{3.4}S_{2}H_{8}+0.6\;CH$$

Equations (34) and (35) show how the CSH and the CH are produced. These two equations have significant importance since they describe the production of CH and CSH. The chemical properties of both CH and CSH control the behavior of cementitious materials in the presence of aggressive environments [54,55].

After segmenting the images using Power’s model Equation (31), three different volume fractions were obtained, capillary pores $\theta_{pores}$, unhydrated cement $\theta_{unhydrated}$, and hydration products $\theta_{hydrated}$, as seen in Figure 7. Each voxel of the image belongs to one of the three groups. Voxels representing unhydrated materials are considered inert. Dissolution can only occur in voxels containing hydrated materials. As mentioned earlier in Section 4.2.1, the images had a resolution of 0.95μm; hence, all features smaller than this value cannot be distinguished. Due to the lack of information on the voxel subscale rather than knowing its chemical composition, several assumptions were made. We assumed here that both CH and CSH are lumped into one phase, the hydrated solid phase. Moreover, both CH and CSH are considered to have the same aqueous saturation concentration of the portlandite $c^{eq}=19.49\;{\mathrm{mol}/m}^{3}$. Hence, we ignored the effect of the silica on the leaching of calcium from CSH.

In order to proceed with the simulation, the saturation concentration of calcium in the hydrated solid materials $c_{Ca,s}^{eq}$ must be known. Since the images give information in the form of grayscale values only, an estimation procedure for $c_{Ca,s}^{eq}$ was adopted. This procedure was based on stoichiometric calculations. In this homogenization procedure, the amount of the CH and the CSH phases forming the hydrated solid materials must be calculated first. A very similar homogenization procedure proposed by Huang et al. [62] was used to extract the effective elastic properties of the HCP from the same CT data sets. The procedure is described in Appendix D. The estimated ratio of calcium saturation concentrations in the pore solution (de-ionized water) and in the hydrated solid phases is then given as $c_{Ca}^{eq}/c_{Ca,s}^{eq}=\frac{19.49\;{\mathrm{mol}/m}^{3}}{26,350.2\;{\mathrm{mol}/m}^{3}}=0.0007396$. This estimated value was very small. Hence, the simulation was expected to run for a long time. Perko and Jacques [63] introduced what is known as the buffer number $Bu=\frac{c^{eq}}{c_{s}}\frac{1}{\theta_{hydrated}}$. The Bu number is a non-dimensional number, which allows increasing $c^{eq}$ or decreasing $c_{s}$ such that “similar dissolution patterns” are obtained after scaling time appropriately according to the relation:

(36) $$t_{2}=t_{1}\frac{Bu_{1}}{Bu_{2}}$$

In the case that $\theta_{hydrated}$ and $c_{s}$ are kept constant and by using the definition of the Bu number, the time is scaled as:

(37) $$t_{2}=t_{1}\frac{c_{1}^{eq}/c_{s}\times \theta_{hydrated}}{c_{2}^{eq}/c_{s}\times \theta_{hydrated}}=t_{1}\frac{c_{1}^{eq}}{c_{2}^{eq}}$$

where $t_{1}$ is the time corresponding to a reference solution saturation concentration $c_{1}^{eq}$ and $t_{2}$ is the scaled time due to using a scaled solution saturation concentration $c_{2}^{eq}$.

Therefore, a system with a specific saturation concentration $c_{reference}^{eq}$ may be accelerated by scaling up its solution saturation concentration to be $c_{accelerated}^{eq}$, which results in having the same morphology obtained at an earlier simulation time, provided that $\theta_{hydrated}$ and $c_{s}$ are kept constant. This acceleration comes with the caveat that $\theta_{hydrated}$ should be more than 10% and Bu should be lower than 0.2 to obtain accurate results [63].

To study the behavior of calcium leaching in the microstructures of HCP, a simulation on the same data set used in the previous section was conducted. As a setup, the sample was surrounded by walls with no-flux boundary conditions, except for the wall at x=0, where a constant zero concentration was prescribed. For the interface between the pores and the anhydrous phase, we also set a no-flux boundary condition. The interface between the hydrated phase and the pores was set to a concentration of $c_{Ca}^{eq}=19.49\;{\mathrm{mol}/m}^{3}$. The simulation was initialized with zero concentration. The resolution was kept the same at $\Delta x=0.95\times 10^{-6}\;m$. The diffusivity of calcium was taken to be $10^{-9}\;m^{2}/s$. The diffusivity in LBM units was $\tilde{D}=100\;\frac{\Delta x^{2}}{\Delta t}$. The time step was then Δt=0.1s. The total number of time steps considered in the simulation was 256,000Δt.

**Figure 9:**
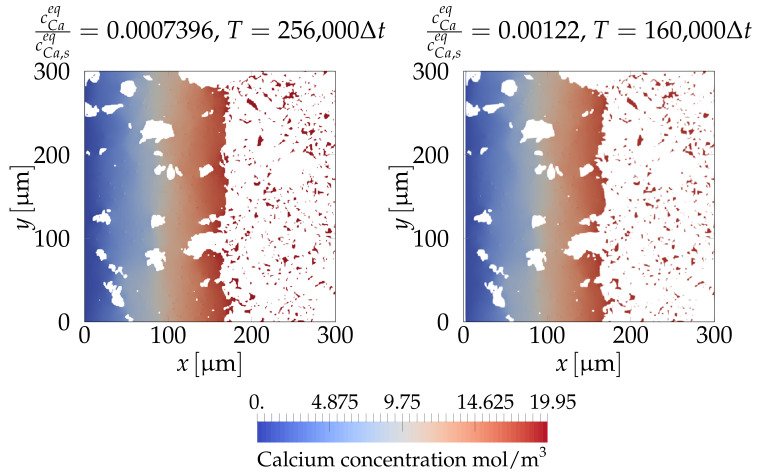
Slices at z=150μm in the calcium concentration field for: the simulation where the original value of the ratio between the saturation concentration in the pore solution and the hydrated solid $c_{Ca}^{eq}/c_{Ca,s}^{eq}=0.0007396$ is employed (**left**) and the simulation of $c_{Ca}^{eq}/c_{Ca,s}^{eq}=0.00122$ where the acceleration approach by [63] is used (**right**). In the first case, we used 256,000 time steps, while in the second case, only 160,000 time steps were used to obtain the results.

Earlier, we calculated $c_{Ca}^{eq}/c_{Ca,s}^{eq}=0.0007396$. The approach of Perko and Jacques [63] was used to arbitrarily increase the ratio $c_{Ca}^{eq}/c_{Ca,s}^{eq}$ by about 60%, to let it become equal to 0.00122. This increase introduced negligible error while at the same time decreased the computational time significantly. To see how the approach of [63] decreased the computational time, Figure 9 shows a comparison of the system evolution for the case of using the original value of $c_{Ca}^{eq}/c_{Ca,s}^{eq}=0.0007396$ and for the case of the accelerated value $c_{Ca}^{eq}/c_{Ca,s}^{eq}=0.00122$. In both simulations, a total number of 256,000 time steps were used. While the original non-accelerated case required all 256,000 time steps for the interface to reach a specific depth, the accelerated approach required a total number of only 160,000 time steps to obtain the same results. It is obvious that the morphology was very similar in both cases and that the leaching depth was almost identical.

**Figure 10:**
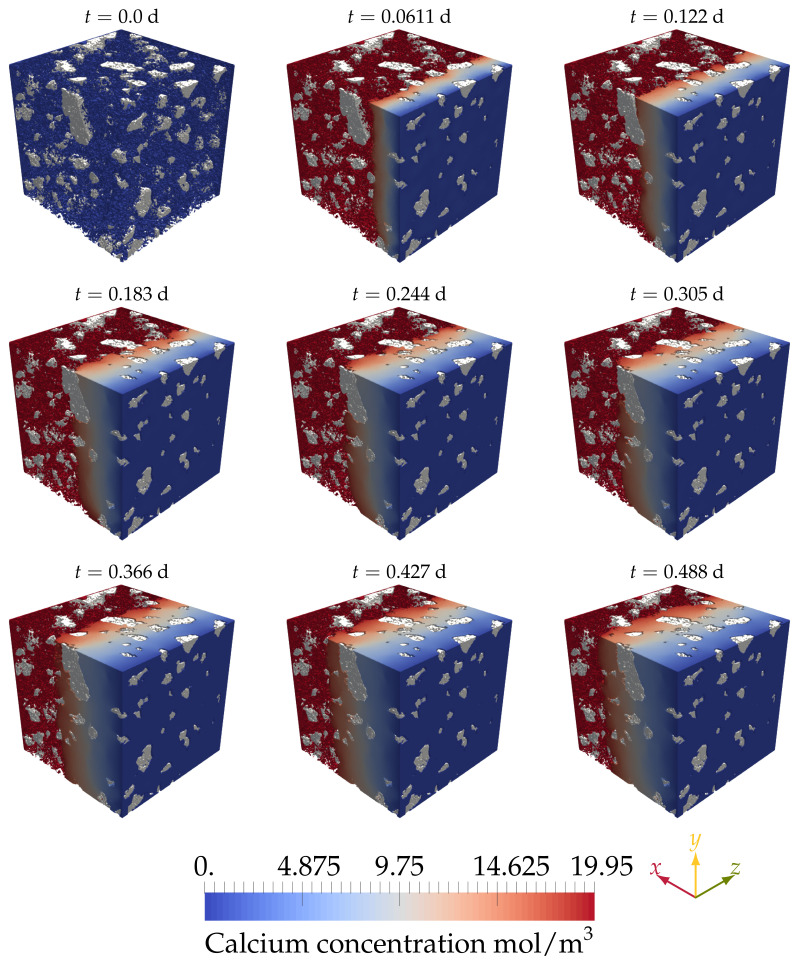
The progression of the calcium leaching front in the HCP. Starting from zero concentration, the pore water reaches its saturation level shortly after the start of the simulation. At the plane x=0, zero concentration was imposed to simulate the existence of an aggressive solution (de-ionized water). The irregular shapes in white are the unhydrated cement particles.

**Figure 11:**
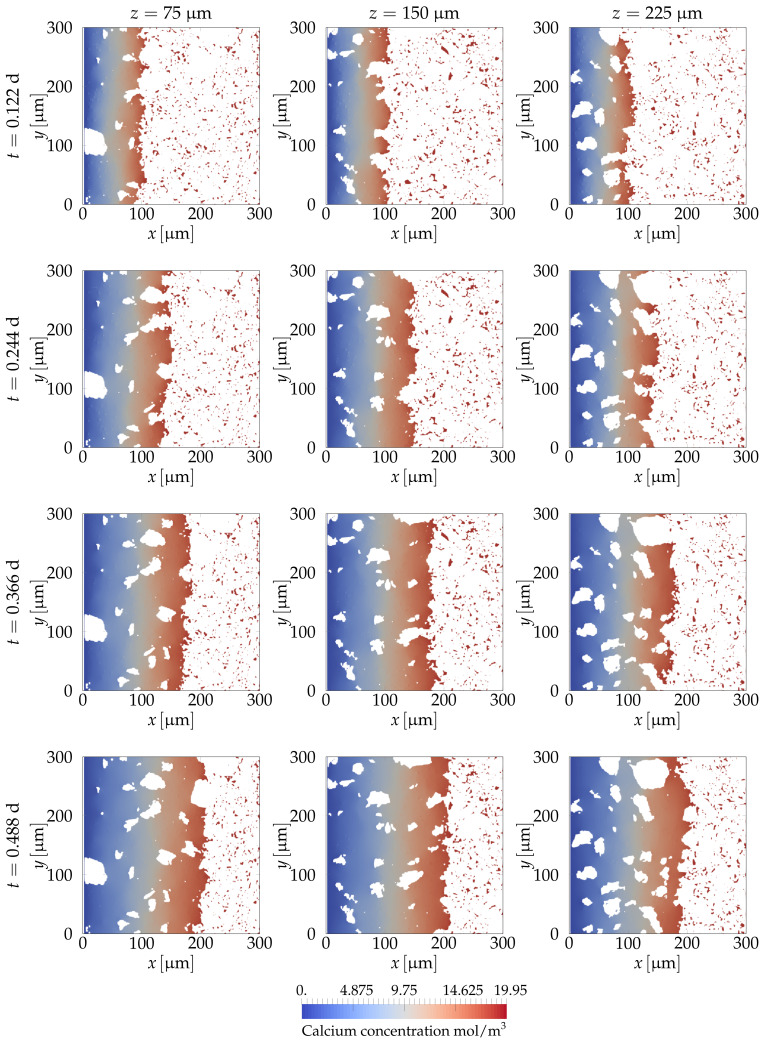
Slices of the dissolution process shown in Figure 10. The columns present slices at planes z∈{75,150,225}μm. The rows of the above figure show the evolution with time of the calcium leaching front. The white regions in the dissolute zone are the locations of the unhydrated cement.

Figure 10 shows the evolution of the system with time. It was observed that at the beginning of the simulation, the concentration in the pores was very far from saturation $c_{Ca}^{eq}=19.49\;{\mathrm{mol}/m}^{3}$. Hence, a surge of calcium mass moved out from the hydrated phases towards the small pores, increasing the concentration to saturation. At the pores nearer to the wall with zero concentration (x=0), the concentration gradient decreased the concentration of calcium at the interface between the hydrated phase and the pores, allowing calcium to dissolve. As time passed, the process continued, and the leaching front moved deeper into the sample. The rate at which the front moved was expected to be proportional to the square root of time past since it is diffusion-controlled. Figure 11 shows different slices taken at different locations and times. It can be seen that dissolution was symmetric along the *y* and *z* directions.

**Figure 12:**
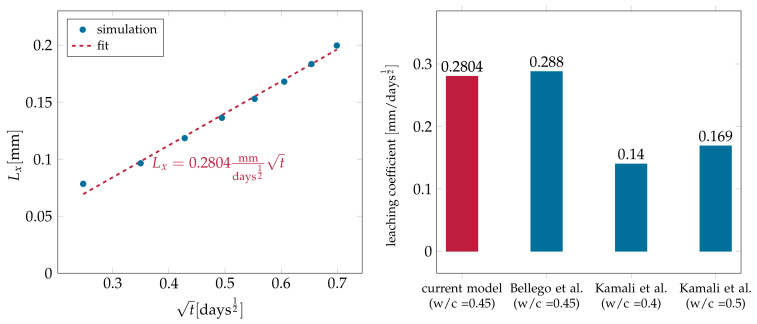
Position of the the average dissolution front together with a fit to obtain the resulting leaching kinetics parameter (**left**) and the comparison to the kinetic parameters from the literature (**right**).

To measure the progression of leaching fronts in hydrated cement pastes in the case of diffusion-controlled dissolution, the depth of leaching is calculated by:

(38) $$L_{x}=a\sqrt{t}$$

where $L_{x}$ is the depth of the leaching front, *a* is known as the leaching kinetics parameter, and *t* is the time. The average position of the interface was calculated and is plotted against the square root of time in Figure 12. Using linear fitting, the leaching kinetics parameter was calculated to be $0.2804\;\mathrm{mm}/{\mathrm{days}}^{\frac{1}{2}}$. A good agreement with Bellego et al. [9,64] can be seen. Bellego et al. investigated the effect of leaching due to deionized water on the mechanical properties of rectangular mortar beams. A similar study, but more general, conducted by Kamali et al. [65], took into account different factors affecting the leaching depth, like the cement composition, admixtures added to the paste, and different temperatures up to 85°C. The specimens used were prisms of size 30 mm × 20 mm × 30 mm. The leaching depth was measured using a scanning electron microscope (SEM). In Figure 12, the leaching kinetics parameter for two of the cases of water/cement w/c∈{0.4,0.5} at $26{\;}^{\circ }C$ were 0.14 and 0.169, respectively.

The deviation from the results of Kamali et al. [65] can be referred to a sum of reasons. We start with the observation of [56,66,67] stating that leaching occurs in stages. That is, portlandite dissolution occurs first, followed by CSH dissolution in an incongruent way. The CSH incongruent dissolution is a function of the calcium-to-silica ratio (Ca/Si) in the solid. It was observed that as Ca/Si decreased, the saturation concentration of calcium in the fluid phase $c_{Ca}^{eq}$ also decreased [56,66,67]. Therefore, in our case, applying homogenization on CH and CSH introduced modeling errors that might have led to this deviation. Distinguishing explicitly between CH and CSH by different boundary conditions would have led to a more accurate model. However, this would require a CT scan in which these two phases can be separated, which was unfortunately not the case for the data available to us. The boundary condition in our simulation assumed a diffusion-controlled dissolution. If the dissolution in the experiment was reaction controlled, it would also explain the overprediction from the simulation, as argued by Varzina et al. [68].

Other factors include the effects of the level of saturation in the pores on transport and the consequences of mechanical stress on transport, which might lead to introducing fractures and microcracks in reality.

### 4.3. Parallel Performance

The performance gain due to the successive segmentation of a job from *r* parallel tasks into *n* parallel tasks with n>r can be measured by taking the ratio between the time required to finish the job in the respective configuration, $T_{r}$ and $T_{n}$, respectively. This ratio is known as the speedup *s*:

(39) $$s=\frac{T_{r}}{T_{n}}$$

Keeping the job size constant while scaling up the number of parallel tasks is referred to as strong scaling. Another indicator for the performance is the parallel efficiency, defined by:

(40) $$E=\frac{s_{m}}{s_{I}}$$

where $s_{m}$ is the measured speedup and $s_{I}=n/r$ is the ideal speedup. A scalability test was conducted on PhoeniX at the Technische Universität Braunschweig, which is a cluster composed of 320 nodes. The standard nodes that were used to run the simulations have 2× CPU INTEL Xeon E5-2640v4 CPUs. Each CPU consists of 20 cores.

In this test, we conducted the performance study directly on the problem found in Section 4.2.3. The block size chosen for the simulation was 19Δx×19Δx×19Δx. The simulation was run for 1000Δt. This is a dynamic problem, where load differs with time. The worst case occurred at the beginning of the simulation where the number of cells containing reactive solid was at a maximum. As time passed, the number of those cells was reduced due to dissolution, and more nodes were converted into fluid. As a result, the overhead of the VOF-PLIC along with the cell updating and node updating algorithms was reduced.

**Figure 13:**
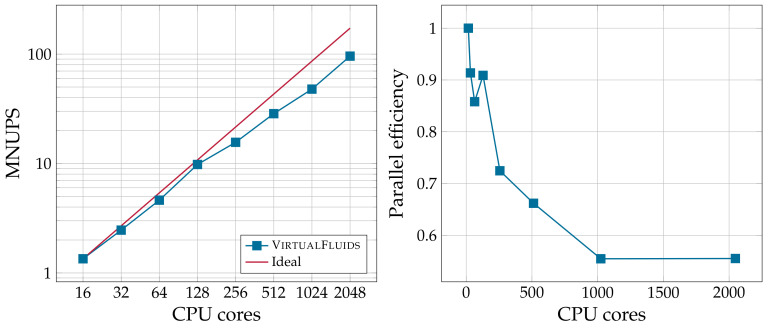
Parallel performance (**left**) and efficiency (**right**) of the FCM together with the VOF-PLIC in simulating the dissolution of calcium from a CT scan of an HCP.

In the LBM, it is customary to measure the performance in terms of the so-called million nodal updates per second (MNUPS). Figure 13 shows the parallel performance on the left and the parallel efficiency on the right. The simulations started with 16 MPI processes due to the fact that pre-processing required a long time. The number of processes was doubled until reaching 2048 MPI processes. It was observed that the parallel performance decreased until reaching a value of 55%.

## 5. Conclusions

In this paper, a framework for the simulation of dissolution of solids was introduced. The framework consisted of a lattice Boltzmann advection-diffusion solver for scalar transport based on the method of factorized central moments (FCMs) and a volume of fluid method, the VOF-PLIC, accounting for morphological changes of the solid body due to dissolution. The FCM method together with appropriate boundary conditions have exceptionally large ranges of admissible transport parameters. This new model was implemented in the massively parallel fluid solver VirtualFluids, which allows tackling problems using large-sized porous media microstructures covering domains larger than a single representative elementary volume, as currently assumed in literature [9]. However, simulating complete macroscopic structures in this approach will remain unfeasible for the foreseeable future. The proposed method can be used to obtain the material properties required for more coarse-grained approaches.

Due to the utilization of off-lattice boundary conditions, a continuous movement of the dissolving interface was obtained with our method despite using a sharp interface. This resulted in second-order accuracy for the position of the interface. In contrast, earlier methods (i.e., [69]) relied on a stochastic update in which the interface jumped in steps of length Δx. The accurate representation of the off-lattice boundary allowed us to compute the normal vector at arbitrary times during the simulation, which is not possible using a stochastic updating.

Continuous movement of the interface can also be realized with diffuse interface models [70]. However, the geometrical flexibility of this approach is limited due to the extended interface required in these methods. Our sharp interface model was based on single node boundary conditions that can deal with corners in all orientations.

The microstructure simulated to obtain the effective diffusivity and the leaching kinetics parameter was directly obtained from a CT scan of a HCP sample. Due to low contrast in the histogram of the CT-scanned cement past, the segmentation into the various solid phases remains difficult. Several approaches may be taken to improve this situation. Among them is the use of advanced segmentation approaches proposed, e.g., by [42,71]. Another alternative to be considered is using machine learning to classify the regions of the scan according to their shape as the shape of hydrated and unhydrated domains is characteristic due to the way they form. Improvements with regards to physics can be applied. For more accurate modeling of the transport, under-saturation conditions are supposed to be taken into account since concrete structures are usually found in under-saturation states in reality. In addition, rather than assuming the CSH phase as an impermeable solid, an accurate model taking into account its incongruent leaching could be developed. Finally, among the major potential improvements is the experimental validation. The fast dynamic processes occurring in the pores was measured only very recently [72]. Therefore, a possible one-to-one comparison of the flow, transport, or morphology change between numerical and experimental results lies on the horizon.

## Figures and Tables

**Table 1:** The positions in the domain where the diffusion velocity $u_{d}=\frac{-D\nabla c}{c}$ reaches the speed of sound $C_{s}\approx 0.577\;\frac{\Delta x}{\Delta t}$.

$\tilde{D}\;\left[\frac{\Delta x^{2}}{\Delta t}\right]$	t(s)	$x\times 10^{-2}\;\left(m\right)$
10,000	72,000	0.4128
10,000	288,000	1.6643
1000	7200	0.4028
1000	28,800	1.6719

**Table 2:** The total sum of the normal to the wall component of the mass flux at the outlet along with the calculated $D_{e}$ for each simulation.

	Along *x* Direction	Along *y* Direction	Along *z* Direction
$\sum j_{i}{|}_{i=300\Delta x}$	10.871	11.036	8.258
$D_{e}/\tilde{D}$	0.0362	0.0367	0.02763
$D_{e}/D_{0}$ (Equation (33))	0.0278		

## Data Availability

The data presented in this study are available on request from the corresponding author.

## Abbreviations

The following abbreviations are used in this manuscript:

BGK	Bhatnagar, Gross, and Krook
CCRL	Cement and Concrete Reference Laboratory
CH	Calcium hydroxide
CSH	Calcium silicate hydrate
CT	Computer tomography
ESRF	European Synchrotron Radiation Facility
FCM	Factorized central moment
HCP	Hydrated cement paste
LBM	Lattice Boltzmann method
MNUPS	Million nodal updates per second
MPI	Message passing interface
MRT	Multiple relaxation time
NIST	National institure of standards and technology
PLIC	Piecewise linear interface construction
REV	Rrepresentative elementary volume
SLIC	Simple line interfac calculation
VOF	Volume of fluid

## Appendix A. From Moments to Distributions and Vice Versa

The forward transformation from distributions to central moments using the velocity of the medium {u,v,w}, which is zero in a pure diffusion problem, reads:

(A1) $$\begin{array}{c} k_{ij|\gamma }=\sum_{k}h_{ijk}{\left(k-w\right)}^{\gamma } \end{array}$$

(A2) $$\begin{array}{c} k_{i|\beta \gamma }=\sum_{j}k_{ij|\gamma }{\left(j-v\right)}^{\beta } \end{array}$$

(A3) $$\begin{array}{c} k_{\alpha \beta \gamma }=\sum_{i}k_{i|\beta \gamma }{\left(i-u\right)}^{\alpha } \end{array}$$

The back transformation from central moments to distributions is:

(A4) $$\begin{array}{ccc} k_{0|\beta \gamma }^{*} & = & k_{0\beta \gamma }^{*}\left(1-u^{2}\right)-2uk_{1\beta \gamma }^{*}-k_{2\beta \gamma }^{*} \end{array}$$

(A5) $$\begin{array}{ccc} k_{\bar{1}|\beta \gamma }^{*} & = & (k_{0\beta \gamma }^{*}\left(u^{2}-u\right)+k_{1\beta \gamma }^{*}\left(2u-1\right)+k_{2\beta \gamma }^{*})/2 \end{array}$$

(A6) $$\begin{array}{ccc} k_{1|\beta \gamma }^{*} & = & (k_{0\beta \gamma }^{*}\left(u^{2}+u\right)+k_{1\beta \gamma }^{*}\left(2u+1\right)+k_{2\beta \gamma }^{*})/2 \end{array}$$

(A7) $$\begin{array}{ccc} k_{i0|\gamma }^{*} & = & k_{i|0\gamma }^{*}\left(1-v^{2}\right)-2vk_{i|1\gamma }^{*}-k_{i|2\gamma }^{*} \end{array}$$

(A8) $$\begin{array}{ccc} k_{i\bar{1}|\gamma }^{*} & = & (k_{i|0\gamma }^{*}\left(v^{2}-v\right)+k_{i|1\gamma }^{*}\left(2v-1\right)+k_{i|2\gamma }^{*})/2 \end{array}$$

(A9) $$\begin{array}{ccc} k_{i1|\gamma }^{*} & = & (k_{i|0\gamma }^{*}\left(v^{2}+v\right)+k_{i|1\gamma }^{*}\left(2v+1\right)+k_{i|2\gamma }^{*})/2 \end{array}$$

(A10) $$\begin{array}{ccc} h_{ij0}^{*} & = & k_{ij|0}^{*}\left(1-w^{2}\right)-2wk_{ij|1}^{*}-k_{i|2\gamma }^{*} \end{array}$$

(A11) $$\begin{array}{ccc} h_{ij\bar{1}}^{*} & = & (k_{ij|0}^{*}\left(w^{2}-w\right)+k_{ij|1}^{*}\left(2w-1\right)+k_{ij|2}^{*})/2 \end{array}$$

(A12) $$\begin{array}{ccc} h_{ij1}^{*} & = & (k_{ij|0}^{*}\left(w^{2}+w\right)+k_{ij|1}^{*}\left(2w+1\right)+k_{ij|2}^{*})/2 \end{array}$$

## Appendix B. Expanding and Inverse Calculation of Equation (18)

In this section, an expanded form of Equation (18) is provided followed by the calculation of the inverse of the same equation. The sorting of the normal components allows writing Equation (18) as a piecewise function with a domain separated into seven main intervals:

(A13) $$V\left(\alpha ,n\right)=\left\{\begin{array}{cc} \frac{\alpha^{3}-\left(\alpha -n_{1}\right){}^{3}}{6n_{1}n_{2}n_{3}} & 0<\alpha \leq n_{1}\\ \frac{\alpha^{3}-\left(\alpha -n_{1}\right){}^{3}-\left(\alpha -n_{2}\right){}^{3}}{6n_{1}n_{2}n_{3}} & n_{1}<\alpha \leq n_{2}\\ \frac{\alpha^{3}-\left(\alpha -n_{1}\right){}^{3}-\left(\alpha -n_{2}\right){}^{3}-\left(\alpha -n_{3}\right){}^{3}}{6n_{1}n_{2}n_{3}} & n_{2}<\alpha \leq \min\left\{n_{1}+n_{2},n_{3}\right\}\\ \left\{\begin{array}{cc} \frac{\alpha^{3}-\left(\alpha -n_{1}\right){}^{3}-\left(\alpha -n_{2}\right){}^{3}-\left(\alpha -n_{3}\right){}^{3}}{6n_{1}n_{2}n_{3}} & n_{3}<\alpha \leq n_{1}+n_{2}\\ \frac{\alpha^{3}-\left(\alpha -n_{1}\right){}^{3}-\left(\alpha -n_{2}\right){}^{3}+\left(\alpha -n_{1}-n_{2}\right){}^{3}}{6n_{1}n_{2}n_{3}} & n_{1}+n_{2}<\alpha \leq n_{3} \end{array}\right. & \min\left\{n_{1}+n_{2},n_{3}\right\}<\alpha \leq \max\left\{n_{1}+n_{2},n_{3}\right\}\\ \frac{\alpha^{3}-\left(\alpha -n_{1}\right){}^{3}-\left(\alpha -n_{2}\right){}^{3}+\left(\alpha -n_{1}-n_{2}\right){}^{3}-\left(\alpha -n_{3}\right){}^{3}}{6n_{1}n_{2}n_{3}} & \max\left\{n_{1}+n_{2},n_{3}\right\}<\alpha \leq n_{1}+n_{3}\\ \frac{\alpha^{3}-\left(\alpha -n_{1}\right){}^{3}-\left(\alpha -n_{2}\right){}^{3}+\left(\alpha -n_{1}-n_{2}\right){}^{3}-\left(\alpha -n_{3}\right){}^{3}+\left(\alpha -n_{1}-n_{3}\right){}^{3}}{6n_{1}n_{2}n_{3}} & n_{1}+n_{3}<\alpha \leq n_{2}+n_{3}\\ \frac{\alpha^{3}-\left(\alpha -n_{1}\right){}^{3}-\left(\alpha -n_{2}\right){}^{3}+\left(\alpha -n_{1}-n_{2}\right){}^{3}-\left(\alpha -n_{3}\right){}^{3}+\left(\alpha -n_{1}-n_{3}\right){}^{3}+\left(\alpha -n_{2}-n_{3}\right){}^{3}}{6n_{1}n_{2}n_{3}} & n_{2}+n_{3}<\alpha \leq n_{1}+n_{2}+n_{3} \end{array}\right.$$

When α reaches one of the critical points in each of the interval constituting the domain of Equation (A13), a critical volume is found. The critical volumes are calculated as follows:

(A14) $$\begin{array}{cccc} V_{1}= & \frac{n_{1}^{2}}{6n_{2}n_{3}} & \; & \alpha =n_{1}\\ V_{2}= & \frac{n_{1}^{2}-3n_{2}n_{1}+3n_{2}^{2}}{6n_{2}n_{3}} & \; & \alpha =n_{2}\\ V_{3}= & \left\{\begin{array}{cc} \frac{n_{1}+n_{2}}{2n_{3}} & n_{1}+n_{2}<n_{3}\\ \frac{\left(n_{1}-n_{3}\right){}^{3}+\left(n_{2}-n_{3}\right){}^{3}+n_{3}^{3}}{6n_{1}n_{2}n_{3}} & \;n_{3}<n_{1}+n_{2} \end{array}\right. & \; & \alpha =\min\{n_{1}+n_{2},n_{3}\}\\ V_{4}= & 1-V_{3} & \; & \alpha =\max\{n_{1}+n_{2},n_{3}\}\\ V_{5}= & 1-V_{2} & \; & \alpha =n_{1}+n_{3}\\ V_{6}= & 1-V_{1} & \; & \alpha =n_{2}+n_{3} \end{array}$$

To calculate α as a function of the volume *V* and the normal n, the inverse of Equation (A13) is required. This can be achieved by solving each sub-function in Equation (A13) for α, which gives:

(A15) $$\alpha \left(V,n\right)=\left\{\begin{array}{cc} \sqrt[{3}]{6n_{1}n_{2}n_{3}V} & 0<V\leq V_{1}\\ \frac{1}{6}\left(\sqrt{72n_{2}n_{3}V-3n_{1}^{2}}+3n_{1}\right) & V_{1}<V\leq V_{2}\\ \mathrm{obtained}\;\mathrm{numerically} & V_{2}<V\leq V_{3}\\ \left\{\begin{array}{cc} \mathrm{obtained}\;\mathrm{numerically} & n_{3}<n_{1}+n_{2}\\ \frac{1}{2}\left(2n_{3}V+n_{1}+n_{2}\right) & n_{1}+n_{2}\leq n_{3} \end{array}\right. & V_{3}<V\leq V_{4}\\ \mathrm{obtained}\;\mathrm{numerically} & V_{4}<V\leq V_{5}\\ -\frac{1}{6}\sqrt{-72n_{2}n_{3}\left(V-1\right)-3n_{1}^{2}}+\frac{n_{1}}{2}+n_{2}+n_{3} & V_{5}<V\leq V_{6}\\ \sqrt[{3}]{6n_{1}n_{2}n_{3}\left(V-1\right)}+n_{1}+n_{2}+n_{3} & V_{6}<V\leq 1 \end{array}\right.$$

where the critical volumes $V_{1}$ to $V_{6}$ are defined in Equation (A14).

## Appendix C. Analysis of Finite Differences for Interface Normal

The computation of the gradient of the volume *V* according to Equation (20) uses an extended stencil with adjustable weights. In this paper, we used the weights proposed by Parker and Youngs [36], which were also successfully used by Janssen et al. [35]. Different weights result in different isotropy properties, as can be seen by the following analysis.

Taylor expanding the value of *V* at the centers of the 27 cells in the stencil and then applying the finite difference operator lead to:

(A16) $$\nabla V=\left(\begin{array}{c} \partial_{x}\left\{W_{1}\left(V+\Delta x^{2}/6\nabla^{2}V\right)-\Delta x^{2}W_{2}\left(\partial_{yy}V+\partial_{zz}V\right)\right\}+O\left(\Delta x^{4}\right)\\ \partial_{y}\left\{W_{1}\left(V+\Delta x^{2}/6\nabla^{2}V\right)-\Delta x^{2}W_{2}\left(\partial_{xx}V+\partial_{zz}V\right)\right\}+O\left(\Delta x^{4}\right)\\ \partial_{z}\left\{W_{1}\left(V+\Delta x^{2}/6\nabla^{2}V\right)-\Delta x^{2}W_{2}\left(\partial_{xx}V+\partial_{yy}V\right)\right\}+O\left(\Delta x^{4}\right) \end{array}\right)$$

with:

(A17) $$\begin{array}{ccc} W_{1} & = & 2w(0,0)+8(w(1,0)+w(1,1)) \end{array}$$

(A18) $$\begin{array}{ccc} W_{2} & = & \frac{w(0,0)}{3}-\frac{2w(1,0)}{3}-\frac{8w(1,1)}{3} \end{array}$$

It is seen that no higher accuracy than second order can be reached with this stencil due to the presence of $\Delta x^{2}/6\nabla^{2}V$. However, as we were only interested in the direction of the gradient, our main concern was the isotropy of the stencil. The finite difference is said to be isotropic to second order if the second-order error of a directional derivative along a vector s, i.e., s·∇V, does not depend on the direction. This is the case for the part multiplied by W1 as $\nabla^{2}V$ is isotropic. However, the second part of the leading error multiplied with $W_{2}$ breaks the isotropy. Therefore, the finite difference is isotropic if $W_{2}=0$. Further, in order to obtain the correct derivative $W_{1}$ must be equal to one. The later is not enforced in practice, as we were only interested in the direction of the derivative, not its magnitude.

Inserting the weights used by Parker and Youngs [36] leads to $W_{2}=-1/12$ such that this finite difference is not isotropic. Using the weights used in the D3Q27 lattice Boltzmann method scaled by a factor of three, i.e., w(0,0)=3×2/27, w(1,0)=3×1/54, and w(1,1)=3×1/216 results in an isotropic finite difference with $W_{2}=0$.

## Appendix D. Homogenization Procedure of the Hydrated Phases

The procedure for calculating the amount of calcium in the CH and the CSH is described in what follows.

The number of moles in one unit volume of cement N$\left[N\;L^{-3}\right]$ for $C_{3}S$ and $C_{2}S$, respectively, can be calculated from:

(A19) $$\begin{array}{cc} N_{C_{3}S} & =\frac{\phi_{C_{3}S}\;\rho_{C_{3}S}}{M_{C_{3}S}}\\ N_{C_{2}S} & =\frac{\phi_{C_{2}S}\;\rho_{C_{2}S}}{M_{C_{2}S}} \end{array}$$

where $M_{C_{3}S}$ and $M_{C_{2}S}$ are the molar masses $\left[M\;N^{-1}\right]$ of $C_{3}S$ and $C_{2}S$, $\rho_{C_{3}S}$ and $\rho_{C_{2}S}$ are the mass densities $\left[M\;L^{-3}\right]$ of $C_{3}S$ and $C_{2}S$, and $\phi_{C_{3}S}$ and $\phi_{C_{2}S}$ are the volume fractions of $C_{3}S$ and $C_{2}S$, respectively.

Since two chemical reactions produce CSH, we distinguish between CSH produced from $C_{3}S$, Equation (34), and the one produced from $C_{2}S$, Equation (35). The number of moles in one unit volume of CSH produced from $C_{3}S$ is $N_{CSH}^{C_{3}S}$, and the number of moles of CSH produced from $C_{2}S$ is $N_{CSH}^{C_{2}S}$:

(A20) $$\begin{array}{cc} N_{CSH}^{C_{3}S} & =\frac{\phi_{CSH}^{C_{3}S}\;\rho_{CSH}}{M_{CSH}}\\ N_{CSH}^{C_{2}S} & =\frac{\phi_{CSH}^{C_{2}S}\;\rho_{CSH}}{M_{CSH}} \end{array}$$

where $M_{CSH}$ is the molar mass of CSH, $\rho_{CSH}$ is the mass density of CSH, $\phi_{CSH}^{C_{3}S}$ is the volume fraction of CSH produced from $C_{3}S$, and $\phi_{CSH}^{C_{2}S}$ is the volume fraction of CSH produced from $C_{2}S$.

The same can be applied to the CH phase:

(A21) $$\begin{array}{cc} N_{CH}^{C_{3}S} & =\frac{\phi_{CH}^{C_{3}S}\;\rho_{CH}}{M_{CH}}\\ N_{CH}^{C_{2}S} & =\frac{\phi_{CH}^{C_{2}S}\;\rho_{CH}}{M_{CH}} \end{array}$$

where $M_{CH}$ is the molar mass of CH, $\rho_{CH}$ is the mass density of CH, $\phi_{CH}^{C_{3}S}$ is the volume fraction of CH produced from $C_{3}S$, and $\phi_{CH}^{C_{2}S}$ is the volume fraction of CH produced from $C_{2}S$.

The stoichiometric ratios between the the products (CSH, CH) to the reactants (C3S, C2S) can be obtained from Equation (34):

(A22) $$\begin{array}{c} \frac{n_{CSH}}{n_{C_{3}S}}=\frac{N_{CSH}^{C_{3}S}}{N_{C_{3}S}}=\frac{1}{2}\\ \frac{n_{CH}}{n_{C_{3}S}}=\frac{N_{CH}^{C_{3}S}}{N_{C_{3}S}}=1.3 \end{array}$$

and Equation (35):

(A23) $$\begin{array}{c} \frac{n_{CSH}}{n_{C_{3}S}}=\frac{N_{CSH}^{C_{2}S}}{N_{C_{2}S}}=\frac{1}{2}\\ \frac{n_{CH}}{n_{C_{2}S}}=\frac{N_{CH}^{C_{2}S}}{N_{C_{2}S}}=0.3 \end{array}$$

where $n_{CSH}$, $n_{CH}$, $n_{C_{3}S}$, and $n_{C_{2}S}$ are the coefficients of Equations (34) and (35).

From Equation (A22), the total number of CSH moles per unit volume at a hydration level α is:

(A24) $$\begin{array}{cc} N_{CSH} & =\alpha (N_{CSH}^{C_{3}S}+N_{CSH}^{C_{2}S})\\ N_{CSH} & =\alpha \left(\frac{1}{2}N_{C_{3}S}+\frac{1}{2}N_{C_{2}S}\right) \end{array}$$

From Equation (A23), the total number of CH moles per unit volume is:

(A25) $$\begin{array}{cc} N_{CH} & =\alpha (N_{CH}^{C_{3}S}+N_{CH}^{C_{2}S})\\ N_{CH} & =\alpha (1.3N_{C_{3}S}+0.3N_{C_{2}S}) \end{array}$$

From Equations (A19) and (A20), we solve for the volume fraction of CSH, $\phi_{CSH}=\phi_{CSH}^{C_{3}S}+\phi_{CSH}^{C_{2}S}$:

(A26) $$\begin{array}{c} \phi_{CSH}=\alpha \frac{M_{CSH}}{\rho_{CSH}}\;\left(\frac{1}{2}N_{C_{3}S}+\frac{1}{2}N_{C_{2}S}\right) \end{array}$$

(A27) $$\begin{array}{c} \phi_{CSH}=\alpha \frac{M_{CSH}}{\rho_{CSH}}\;\left(\frac{1}{2}\frac{\phi_{C_{3}S}\;\rho_{C_{3}S}}{M_{C_{3}S}}+\frac{1}{2}\frac{\phi_{C_{2}S}\;\rho_{C_{2}S}}{M_{C_{2}S}}\right) \end{array}$$

The same procedure is done to obtain the volume fraction of CH, $\phi_{CH}=\phi_{CH}^{C_{3}S}+\phi_{CH}^{C_{2}S}$:

(A28) $$\begin{array}{c} \phi_{CH}=\alpha \frac{M_{CH}}{\rho_{CH}}\;\left(1.3N_{C_{3}S}+0.3N_{C_{2}S}\right) \end{array}$$

(A29) $$\begin{array}{c} \phi_{CH}=\alpha \frac{M_{CH}}{\rho_{CH}}\;\left(1.3\frac{\phi_{C_{3}S}\;\rho_{C_{3}S}}{M_{C_{3}S}}+0.3\frac{\phi_{C_{2}S}\;\rho_{C_{2}S}}{M_{C_{2}S}}\right) \end{array}$$

On the level of cement paste, the volume fractions for both CSH and CH:

(A30) $$\begin{array}{c} \theta_{CSH}=\phi_{hydrated}\frac{\phi_{CSH}}{\phi_{CH}+\phi_{CSH}} \end{array}$$

(A31) $$\begin{array}{c} \theta_{CH}=\phi_{hydrated}\frac{\phi_{CH}}{\phi_{CH}+\phi_{CSH}} \end{array}$$

According to Taylor [73], the densities can be estimated to be $\rho_{C_{3}S}=3150\;{\mathrm{kg}/m}^{3}$, $\rho_{C_{2}S}=3280\;{\mathrm{kg}/m}^{3}$, and $\rho_{CH}=2240\;{\mathrm{kg}/m}^{3}$. Huang et al. [62] gave the density of CSH as $\rho_{CSH}=1824.59\;{\mathrm{kg}/m}^{3}$. Bernard et al. [74] gave values for the molar masses: $M_{C_{3}S}=228.32\;g/\mathrm{mol}$, $M_{C_{2}S}=172.24\;g/\mathrm{mol}$, $M_{CH}=74\;g/\mathrm{mol}$, and $M_{CSH}=227.2\;g/\mathrm{mol}$.

The physical properties of the reactants and products were used together with the volume fractions of the reactants from $\phi_{C_{3}S}=0.7018$, $\phi_{C_{2}S}=0.1315$, $\phi_{C_{3}A}=0.0827$, and $\phi_{C_{4}AF}=0.084$ provided by NIST [43,44] to calculate $\theta_{CH}$ and $\theta_{CSH}$. Both were estimated to be 0.204 and 0.35173, respectively. Their sum was equal to the value of $\theta_{hydrated}$. Hence, one cubic meter of the hydration products was split into 36.708% CH and 63.29% CSH. From this, we can calculate the saturation concentration of calcium in the hydration products:

$$\begin{array}{cc} c_{Ca,\;s}^{eq}=\; & 0.36708\;\times \frac{2240}{74\times 10^{-3}}\left[\frac{\mathrm{mol}\;\mathrm{CH}}{m^{3}}\right]\times 1\left[\frac{\;\mathrm{mol}\;\mathrm{Ca}}{\;\mathrm{mol}\;\mathrm{CH}}\right]+\\ \; & 0.6329\;\times \frac{1824.59}{227.2\times 10^{-3}}\left[\frac{\mathrm{mol}\;\mathrm{CSH}}{m^{3}}\right]\times 3\left[\frac{\;\mathrm{mol}\;\mathrm{Ca}}{\;\mathrm{mol}\;\mathrm{CSH}}\right]=26350.2\left[\frac{\mathrm{mol}}{m^{3}}\right] \end{array}$$
